# 800 fps neutron radiography of air-water two-phase flow

**DOI:** 10.1016/j.mex.2018.01.008

**Published:** 2018-01-31

**Authors:** Robert Zboray, Pavel Trtik

**Affiliations:** a Laboratory of Thermal Hydraulics, Division of Nuclear Energy and Safety, Paul Scherrer Institut, 5232 Villigen PSI, Switzerland; b Laboratory for Neutron Scattering and Imaging, Paul Scherrer Institut, 5232 Villigen PSI, Switzerland

**Keywords:** Neutron radiography, Neutron imaging, Dynamic imaging, Bubbly flow, Two-phase flow

## Abstract

We have demonstrated dynamic cold neutron imaging of air-water two-phase flows up to 800 frames per second imaging rates. This has been achieved by using a high-efficiency (relatively thick) scintillator screen in combination with the highest available flux on a continuous spallation source and a high-speed sCMOS camera. This combination renders the spatial resolution to relatively modest value of about 0.5 mm, which is nevertheless sufficient for resolution of bubbles of the size down to about 1.0 mm in motion with unprecedented framerate using neutron imaging. We show the feasibility of the technique on the two-phase flow at ambient temperature and atmospheric pressure conditions, with the foreseen aim of measurements of two phase flows at high-temperatures and high pressures. It is also foreseen that the technique will be further utilized for quantification of the time-resolved instantaneous gas fraction and the gas phase velocity.

• Demonstration of up to 800 frames per second dynamic cold neutron radiography.
• Application of such technique for non-periodic (transient) process of bubbly flow in water.
• Potential for quantification of (i) instantaneous gas volume fraction in dynamic two-phase flow and (ii) instantaneous gas phase velocimetry.

## Graphical abstract

**Figure:**
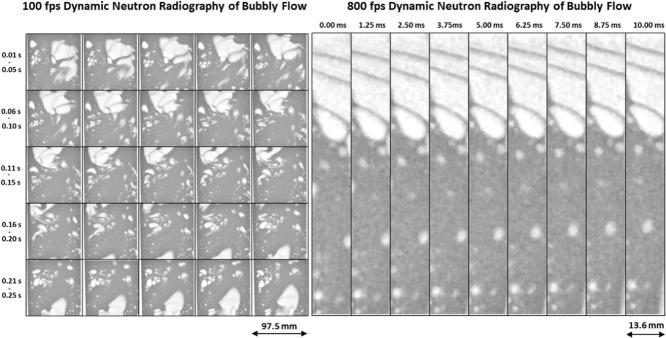

## Method details

Gas-liquid two-phase flows play an important role in many scientific and engineering problems. These include the efficient and safe operation of chemical reactors, heat exchangers, boilers and nuclear reactors just to mention a few. Two-phase flows, in general, are very rapidly changing processes, requiring high temporal resolution instrumentation techniques to capture their dynamics. Neutron imaging being a non-intrusive, non-contact technique can provide many advantages for investigating two-phase flows. In particular, heated, boiling two-phase flows under high-temperature, high-pressure conditions enclosed in thick metallic containers for mechanical stability could be a very interesting subject of non-intrusive investigation by neutron imaging. Neutrons can easily penetrate heavy metallic materials while being sensitive for even small amounts of water while photon-based techniques just possess opposite features therefore being much less, if at all, feasible.

It is the recent developments in sCMOS detector technology that allow neutron imaging of fast processes with high temporal resolution. The negligible readout-time and low read-out noise of sCMOS cameras allow for the “continuous” observation of non-cyclic processes with high-temporal resolution. A few examples of high temporal resolution neutron imaging of industrially relevant samples utilizing the sCMOS technology include: the visualization of flows in liquid metals with acquisition time of approximately 0.03 s in 2D [1], and on-the-fly tomography of water uptake in roots [2]. An overview of the high-temporal resolution imaging for studies of porous media has been recently published by Kaestner et al. [3]. Nakamura et al., investigated oil behavior in a small 4 cycle engine at 30 fps [4]. Murakawa et al., developed a dynamic neutron tomography with 15 s repetition rate [5]. Note that neutron imaging at even higher temporal resolution (with exposure times down to 50 μs) has hitherto been available only for the case of repetitive/cyclic processes (e.g. imaging of running motors) using an ensemble averaged phase-lock modality of the neutron imaging [6]. This is mainly due to the fact that the available neutron flux even at the most advanced neutron sources (spallation or reactor-based) world-wide is limited (typically in the range of few times 10^7^ to few times 10^8^ neutrons/s/cm^2^). Such values translate to a limited number of captured neutrons for a 100 × 100 μm pixel per 0.01 s acquisition time. Therefore, especially for non-periodic processes, one has to usually compromise the spatial resolution to enable high temporal resolution. One of the possible ways to work around the limited flux problem is to use focusing neutron guides to maximize locally the available flux. This has been proven by Trtik et al. [7], who investigated mixing of heavy and light water at frame rates of 100 frames per second (fps) with high signal to noise ratio. Nevertheless, one has to compromise the available field-of-view (FOV) with high intensity around the focal spot of the guide.

Regarding neutron imaging of highly dynamic, non-periodic air-water two-phase flows imaging rates up to 300 fps were achieved for air-water two-phase flows using fast neutrons at the a high-intensity, accelerator-based neutron facility of the Physikalisch Technische Bundesanstalt (PTB), Germany [8]. Such frame rates are necessary to resolve highly dynamic two-phase flows without significant motion artifacts (see below). A special detector incorporating among others a thick, plastic scintillator fiber screen and an image intensifier has been used at a moderate spatial resolution of 1.75 mm with a fast neutron flux in the aforementioned range.

In the present investigations, we aimed at even higher image capture rates using cold neutrons at the ICON imaging beam line of the continuous spallation neutrons source SINQ, Switzerland [9] using a combination of the maximal available flux through the largest beam aperture and a high-speed sCMOS camera; and making a reasonable compromise on the available space resolution using pixel binning to improve counting statistics for short exposure times. Bubbly two-phase flows have been imaged by thermal neutrons and a flat panel detector at NIST [10] at a low rate of 30 fps, Hillenbach et al. [11], imaged boiling of pentane in a steel tube at 100 fps. The provided neutron movie of the process clearly shows the strong influence of the motion artifacts at such imaging rate.

## Test arrangement

The flat bubbler (with internal dimensions of 300 mm in height, 98 mm in width but of only 5 mm depth in beam direction) filled with tap water has been imaged placed directly in front of the detector screen to minimize geometrical blur. The two-phase flow was produced by injecting air through an air inlet (4 mm ID) placed at 95 mm below the FOV in the center line of the bubbler. The flat bubbler of the above dimensions is made of aluminum of 4 mm wall thickness. The water was not recirculated externally, however due to the drag force by the air bubbles internal water recirculation was generated. A 300 μm thick, ^6^LiF/ZnS(Cu) scintillator screen has been used to maximize the detection efficiency while achieving reasonable light output. A Hamamatsu ORCA Flash 4.0 high-speed sCMOS camera with 16 bit color depth in a MIDI-box setup [11] was used for the imaging in combination with a 50 mm Nikon AF-S NIKKOR 1:1.4 lens. The unbinned pixel size of such test arrangement was equal to 53 μm. To improve counting statistics for the projected short exposures, a 4 × 4 pixel binning has been utilized directly on the camera reducing the FOV to 512 × 512 pixels, thus resulting in pixel size of 212 μm. Therefore the full FOV equaled about 108 × 108 mm × mm. The applied image frame rates were 100 and 800 fps, respectively for all experiments. It needs to be highlighted here that due to nature of the sCMOS readout, only 100 fps radiographies can utilize the full FOV of 512 × 512 pixels (100 fps full frames being the constant readout speed of a camera transmitting the data directly to a PC via a Camera Link performed continuously). The radiographies of 800 fps rates are limited by a reduced FOV in the horizontal direction by a factor of 8 to 64 pixels only, centered on the image mid vertical axis. The higher acquisition rate corresponds to a proportionally lower images size, rendering the image storage requirements the same and producing typically around 0.5 GB data in a 10 s long run.

The imaging has been performed at the ICON beamline [9]. In order to achieve the highest available neutron flux the aperture of 80 mm in diameter was used resulting in a neutron flux of approximately 2 × 10^8^ n/(cm^2^ s) at the sample position. The detector was positioned at 7630 mm distance from the aperture and therefore the resulting L/D ratio was equal to approximately 95. The use of a relatively thick scintillator screen resulted in a modest spatial resolution of about 500 μm, which has been determined by imaging a Gd Siemens Star patterns placed on the scintillator screen as is described in [12].

The resulting series of neutron radiographies were analyzed in the following manner. Each radiograph was corrected for open beam and dark current image (each based on median value of at least 100 images). Next, the scarcely occurring gamma (white spots) were suppressed using remove outliers routine in ImageJ [13]. The noise in the resulting radiographs was then suppressed using anisotropic diffusion filtering procedure [14] implemented for ImageJ by Műnch [15,16]. Note that the contrast-to-noise ratio between water and air in the bubbler was typically around 17.7 at 100 fps and –as can be expected due to diminishing signal-to-noise ratio- much lower but still reasonably high, 5.5 at 800 fps.

## Results and discussion

**Fig. 1:**
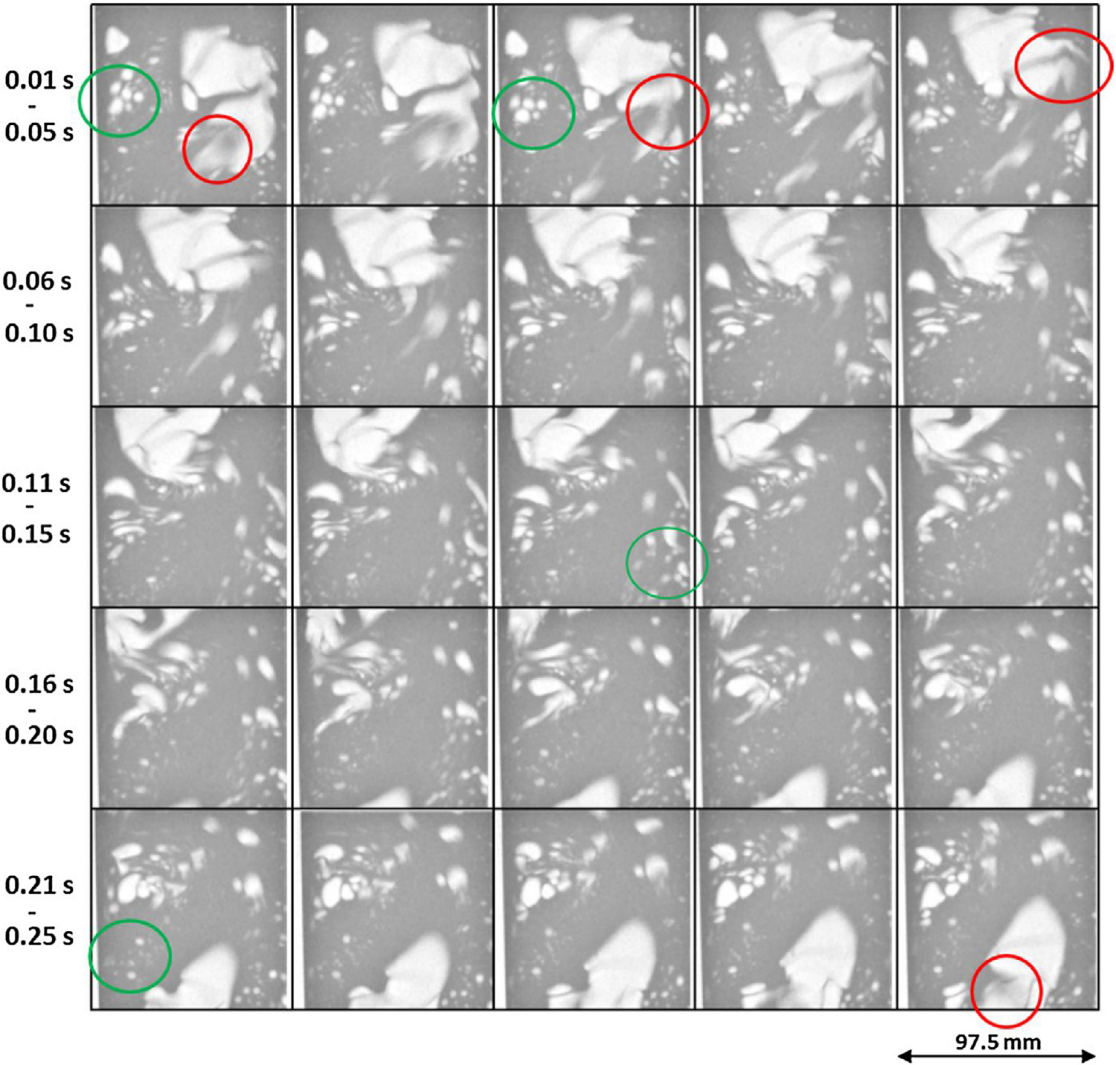
Twenty five subsequent images of 100 fps neutron imaging experiment showing the bubbly flow of air in H_2_O. Please note that 100 fps is not sufficient temporal resolution for observation of the fast moving (relatively large) bubbles (see few of those cases highlighted by a red ellipses), while the slowly moving predominantly smaller bubbles (see few highlighted in a green ellipses) are visualized sharply.

**Fig. 2:**
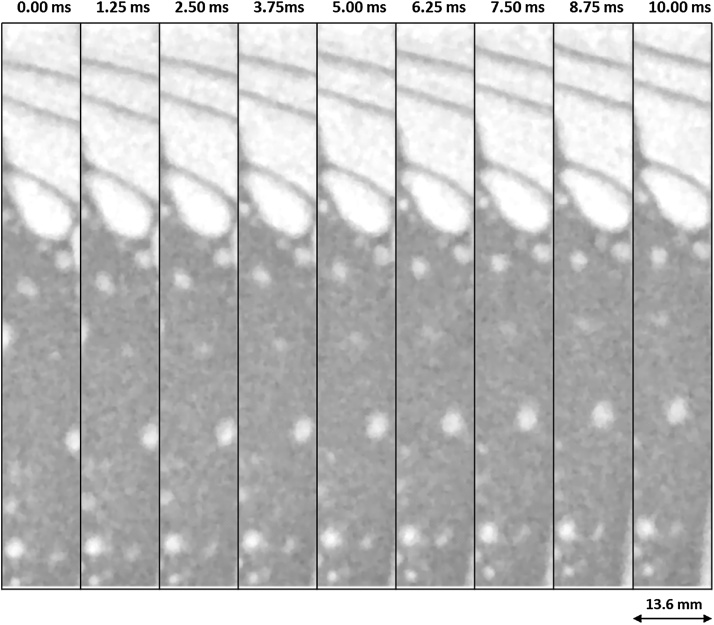
Nine subsequent images of 800 fps neutron imaging experiment showing the bubbly flow of air in H_2_O. This frame rate is sufficient for resolving the interfaces between fast moving large accelerating bubbles (in the top of the respective images).

**Fig. 3:**
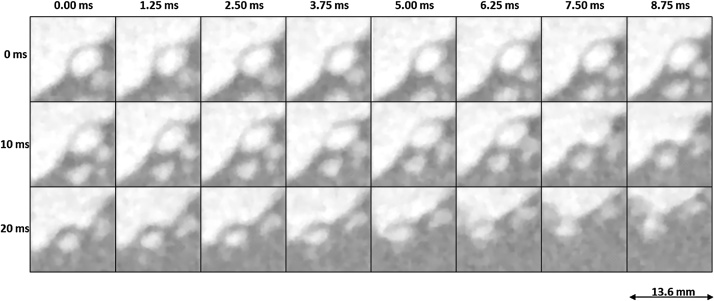
Twenty four subsequent images of 800 fps neutron imaging experiment showing the area of interest in which two bubbles (one larger than the available field of view) coalesce.

The images in Fig. 1 show the bubbly flow using 100 fps acquisition rate. This frame rate is clearly not sufficient for visualization of the bubbly flow, as the fast moving (usually the large) bubbles are visualized rather blurred (see e.g. the in red highlighted large bubbles in Fig. 1). The phenomena of bubble coalescence and break up can be very well observed in detail using the high temporal resolution radiographies at 800 fps (see Fig. 2, Fig. 3 and corresponding .avi files in Supplementary materials). Also, the liquid interfaces between very closely spaced bubbles (that are either coalescing or breaking up) are well resolved at 800 fps. Depending on the instantaneous speed of the individual bubbles, the visualization of these can be significantly blurred (in particular for the accelerating bubbles). The images of the bubbles become sharper again upon bubble deceleration (see movie in the Supplementary material – “Insert Link”).

As the longer radiographic series were actually captured, an .avi file showing the video of the extended version of this image sequence is available in the Supplementary materials of this paper (Appendix A).

It should be noted that bubbles of a several millimeters size rise in quiescent water at typical velocities around 0.5 m/s resulting in a blur of about 5 mm due to motion artifacts at e.g. 100 fps. This would give together with the motionless spatial resolution of 0.5 mm an effective spatial resolution for the dynamic imaging of $\sqrt{5^{2}+0.5^{2}}=5.02\;mm$ clearly dominated by the motion blur. Therefore it is important to maximize the imaging rate to decrease this, otherwise only large bubbles (>5 mm) can be time-resolved with relatively low blurring. Still considering the 0.5 m/s bubble velocity, the effective spatial resolution at 800 fps frame rate equals $\sqrt{0.625^{2}+0.5^{2}}=0.8\;\;mm$ and is already very close to the motionless value. Note that due to the internal water recirculation in our bubbler causing continuous deceleration and acceleration, the instantaneous bubble velocities might be differing time-to-time considerably from the above value.

The results have the potential to enable quantitative determination of the time-resolved instantaneous gas fraction and the gas phase velocity which will be demonstrated in a paper. Nevertheless, just to put the results presented here into context with respect to potential applications, we should mention that actual bubbly flow velocities in e.g. bubble column reactors (chemical industry) or in bioreactors are typically very close to that we had in our bubbler. For boiling two-phase flow in high-power heat exchangers or in nuclear fuel bundles are typically in the range of several m/s, correspondingly frame rates of several kfps would be required to image such flows at prototypical conditions with minimal motion blur. However imaging boiling flow mockups at reduced scales and velocities corresponding to our present frame rate, would also provide extremely useful data on the flow dynamics for the validation of computational codes used to model such systems.

Likewise, it should be highlighted that the used scintillator screen was not optimized for the purpose of the high-temporal resolution neutron imaging either. Two aspects of the scintillator screens could be optimized in the future for reducing eventual bias effects for quantitative analysis, namely, the neutron capture efficiency of the scintillator should be increased, while at the same time the light output decay time should be suppressed as much as possible. Regarding the latter it should be however noted that Hillenbach et al. [11], using a similar ZnS-based scintillator point out that the scintillation light emission decays below 10% in 85 us, therefore the afterglow is likely to have only minor effect at our imaging rates.

It should be noted here that the imaging frame rate could be further increased likely above 1k fps utilizing a higher flux neutron beam line such as Antares at the FRM-II reactor [17], at the ILL reactor [18] or at the imaging beam line ODIN of the future European Spallation Source (ESS) [19].

The present investigations proved the feasibility of the technique to resolve two-phase flows at time scales that enable investigating the dynamics of processes relevant for practical applications. Furthermore from the prospective application of the technique for heated, boiling two-phase flows many technical and scientific domains could significantly profit. The data available can shed light on the dynamics of convective boiling processes non-intrusively and at unprecedented quality that would be difficult or unfeasible to observe by other methods.

## Conclusion

This short paper presents the results of high temporal resolution cold neutron radiography on highly dynamic air-water two-phase flows using an efficient scintillator screen with a sCMOS camera. We show that 800 fps imaging rate is achievable at very reasonable image quality utilizing the available flux of the SINQ spallation neutron source. The image quality at 800 fps clearly allows tracking the dynamics of bubble coalescence and break up phenomena on few millimeter sized bubbles sharply resolving the interface between them in motion. We demonstrate the significant reduction of motion blur from 100 fps to 800 fps imaging rate. The data are of sufficient quality to be further analyzed for the quantitative evaluation of the instantaneous gas volume fraction and instantaneous gas phase velocities.

## References

[1] Sarma M., Ščepanskis M., Jakovičs A., Thomsen K., Nikoluškins R., Vontobel P., Beinerts T., Bojarevičs A., Platacis E. (2015). Neutron radiography visualization of solid particles in stirring liquid metal. Phys. Proced..

[2] Zarebanadkouki M., Carminati A., Kaestner A., Mannes D., Morgano M., Peetermans S., Lehmann E., Trtik P. (2015). On-the-fly neutron tomography of water transport into lupine roots. Phys. Proced..

[3] Kaestner A., Trtik P., Zarebanadkouki M., Kazantsev D., Snehota M., Dobson K., Lehmann E. (2016). Recent developments in neutron imaging with applications for porous media research. Solid Earth.

[4] Nakamura M., Sugimoto K., Asano H., Murakawa H., Takenaka N., Mochiki N. (2009). Visualization of oil behavior in a small 4-cycle engine with electrical motoring by neutron radiography. Nucl. Instrum. Methods Phys. Res. Sect. A.

[5] Murakawa H., Hashimoto M., Sugimoto K., Asano H., Takenaka N., Mochiki K., Yasuda R. (2011). Development of a dynamic CT system for neutron radiography and consecutive visualization of three-dimensional water behavior in a PEFC stack. Trans. Jpn. Soc. Mech. Eng. Ser. B.

[6] Grűnzweig C., Mannes D., Kaestner A., Schmid F., Vontobel P., Hovind J., Hartmann S., Peetermans S., Lehmann E. (2013). Progress in industrial applications using modern neutron imaging techniques. Phys. Proced..

[7] Trtik P., Morgano M., Bentz R., Lehmann E. (2016). 100 Hz neutron radiography at the BOA beamline using a parabolic focusing guide. MethodsX.

[8] Zboray R., Dangendorf V., Mor I., Bromberger B., Tittelmeier K. (2015). Time-resolved fast-neutron radiography of air-water two-phase flows in a rectangular channel by an improved detection system. Rev. Sci. Inst..

[9] Kaestner A.P., Hartmann S., Kühne G., Frei G., Grünzweig C., Josic L., Schmid F., Lehmann E.H. (2011). The ICON beamline – a facility for cold neutron imaging at SINQ. Nucl. Instrum. Methods Phys. Res. Sect. A.

[10] NIST (2018). Bubbler Experiments. https://www.nist.gov/video/asibubbles.

[11] Hillenbach A., Engelhardt M., Abelea H., Gahler R. (2005). High flux neutron imaging for high-speed radiography, dynamic tomography and strongly absorbing materials. Nucl. Instrum. Methods Phys. Res. Sect. A.

[12] Grűnzweig C., Frei G., Lehmann E., Kühne G., David C. (2007). Highly absorbing gadolinium test device to characterize the performance of neutron imaging detector systems. Rev. Sci. Instrum..

[13] Schneider C.A., Rasband W.S., Eliceiri K.W. (2012). IH Image to ImageJ: 25 years of image analysis. Nat. Methods.

[14] Perona P., Malik J. (1990). Scale-space and edge detection using anisotropic diffusion. IEEE Trans. Pattern Anal. Mach. Intell..

[15] B. Műnch, http://imagej.net/Xlib.

[16] Trtik P., Műnch B., Gasser P., Leemann A., Loser R., Wepf R., Lura P. (2011). Focussed ion beam nanotomography reveals the 3D morphology of different solid phases in hardened cement pastes. J. Microsc..

[17] Tremsin A.S., Dangendorf V., Tittelmeier K., Schillinger B., Schulz M., Lerche M., Feller W.B. (2015). Time-resolved neutron imaging at ANTARES cold neutron beamline. JINST.

[18] Ageron P. (1989). Cold neutron sources at ILL. Nucl. Instrum. Methods Phys. Res. Sect. A.

[19] Morgano M., Lehmann E., Strobl M. (2015). Detectors requirements for the ODIN beamline at ESS. Phys. Proced..

